# Missed Foreign Body Aspiration: Fentanyl Patch Leading to Severe Pneumonia and Empyema

**DOI:** 10.7759/cureus.77931

**Published:** 2025-01-24

**Authors:** Mena Louis, Rafael Tapia, Nathaniel Grabill, Navneeth Bongu, Hardeep Singh, J Clifton Hastings

**Affiliations:** 1 General Surgery, Northeast Georgia Medical Center Gainesville, Gainesville, USA; 2 Surgery, Northeast Georgia Medical Center Gainesville, Gainesville, USA; 3 Pulmonary and Critical Care, Northeast Georgia Medical Center Gainesville, Gainesville, USA; 4 Research, Northeast Georgia Medical Center Gainesville, Gainesville, USA; 5 Cardiothoracic Surgery, Northeast Georgia Medical Center Gainesville, Gainesville, USA

**Keywords:** bronchoscopy, chronic substance abuse, empyema, fentanyl patch, foreign body aspiration, pleural effusion

## Abstract

Empyema, defined as a purulent pleural effusion within the pleural cavity, typically results from severe infections and presents significant diagnostic and therapeutic challenges. This case report details the intricate clinical journey of a 44-year-old male with a complex medical history, including chronic substance abuse, significant tobacco use, and untreated hepatitis C, who presented with acute respiratory failure, hypoxia, and right-sided chest pain. Despite initial management with antibiotics and chest tube drainage, the patient's condition necessitated advanced surgical intervention due to persistent sanguineous output.

A right thoracoscopy converted to open thoracotomy with decortication was performed due to dense adhesions. Intraoperatively, bronchoscopy revealed a hidden fentanyl patch in the right lower lobe, which was removed, leading to a significant release of purulent material and marked improvement in the patient’s condition. Postoperatively, the patient steadily recovered with decreased oxygen requirements and improved ambulation. However, he developed acute tubular necrosis, likely due to nephrotoxic medications, which was managed appropriately.

The patient was discharged with instructions for pulmonary function tests and lifestyle modifications, including cessation of tobacco use. This case emphasizes the importance of considering foreign body aspiration in patients with persistent or unexplained pulmonary symptoms, especially those with a history of substance abuse or unexplained recurrent respiratory infections. The multidisciplinary approach, comprehensive diagnostics, and collaborative care were pivotal in achieving a successful outcome, offering valuable insights for similar future cases.

## Introduction

Empyema often arises as a complication of parapneumonic effusion, requiring prompt and effective management to prevent progression to severe complications [1-3]. The standard approach to managing empyema includes antibiotic therapy, drainage procedures such as chest tube placement, and, when necessary, fibrinolytic therapy using agents like tissue plasminogen activator (tPA) and dornase alfa, which are highly supported in the literature [4]. In more severe cases, surgical interventions, including thoracoscopic or open thoracotomy for pleural washout and decortication, may be required to fully evacuate the infected material and restore lung function [5]. This case report focuses on the intricate management of a right loculated pleural effusion (empyema) complicated by parapneumonic effusion and the unexpected discovery of a fentanyl patch in the right lower lobe.

The presence of a foreign body within the airway, such as a fentanyl patch, is an unusual finding that can significantly affect the clinical course and treatment strategy [6]. Foreign body aspiration is more commonly recognized in pediatric populations but remains a challenging diagnosis in adults [7]. This mechanical obstruction can lead to persistent post-obstructive pneumonia and inflammatory responses, potentially resulting in pneumonia and empyema [8].

The workup for empyema generally begins with imaging studies, such as chest X-rays and computed tomography (CT) scans, to confirm the presence of pleural fluid and identify any loculations [9]. If clinical and radiological findings strongly suggest empyema, early drainage is often pursued to achieve source control while obtaining fluid for analysis and culture. In cases where the diagnosis is not certain, diagnostic thoracentesis can help distinguish between an uncomplicated parapneumonic effusion and empyema [10]. Laboratory tests on the pleural fluid-such as pH, glucose, lactate dehydrogenase (LDH), and microbiological cultures-provide critical information to guide treatment decisions [6].

Management of empyema encompasses both medical and surgical interventions [11]. Initial treatment includes broad-spectrum antibiotics tailored to culture results [12]. Drainage of the pleural space is crucial and can be accomplished through chest tube placement, often supplemented with fibrinolytic therapy using agents such as tPA and dornase alfa to break down loculations and enhance fluid drainage [13]. In more advanced cases, surgical procedures such as video-assisted thoracoscopic surgery (VATS) or open thoracotomy with decortication are employed to remove the thickened pleural peel, facilitate lung re-expansion, and eliminate any residual potential space that could sustain ongoing infection [14].

## Case presentation

The patient is a 44-year-old male with a notable medical history that includes chronic substance abuse, peripheral neuropathy, chronic productive cough, and untreated hepatitis C diagnosed four years before presentation. He accumulated a 40-pack-year smoking history (two packs per day for 20 years) but has since quit. He also has a family history of lung cancer and emphysema.

The patient presented to the hospital with acute respiratory failure, hypoxia, and right-sided chest pain, reporting that his symptoms had progressively worsened over the past few days. Earlier in the year, he had been treated for walking pneumonia. On arrival, he exhibited a high fever (Tmax 103.2°F), tachycardia, and persistent right-sided chest pain. Physical examination revealed decreased breath sounds on the right side.

**Figure 1:**
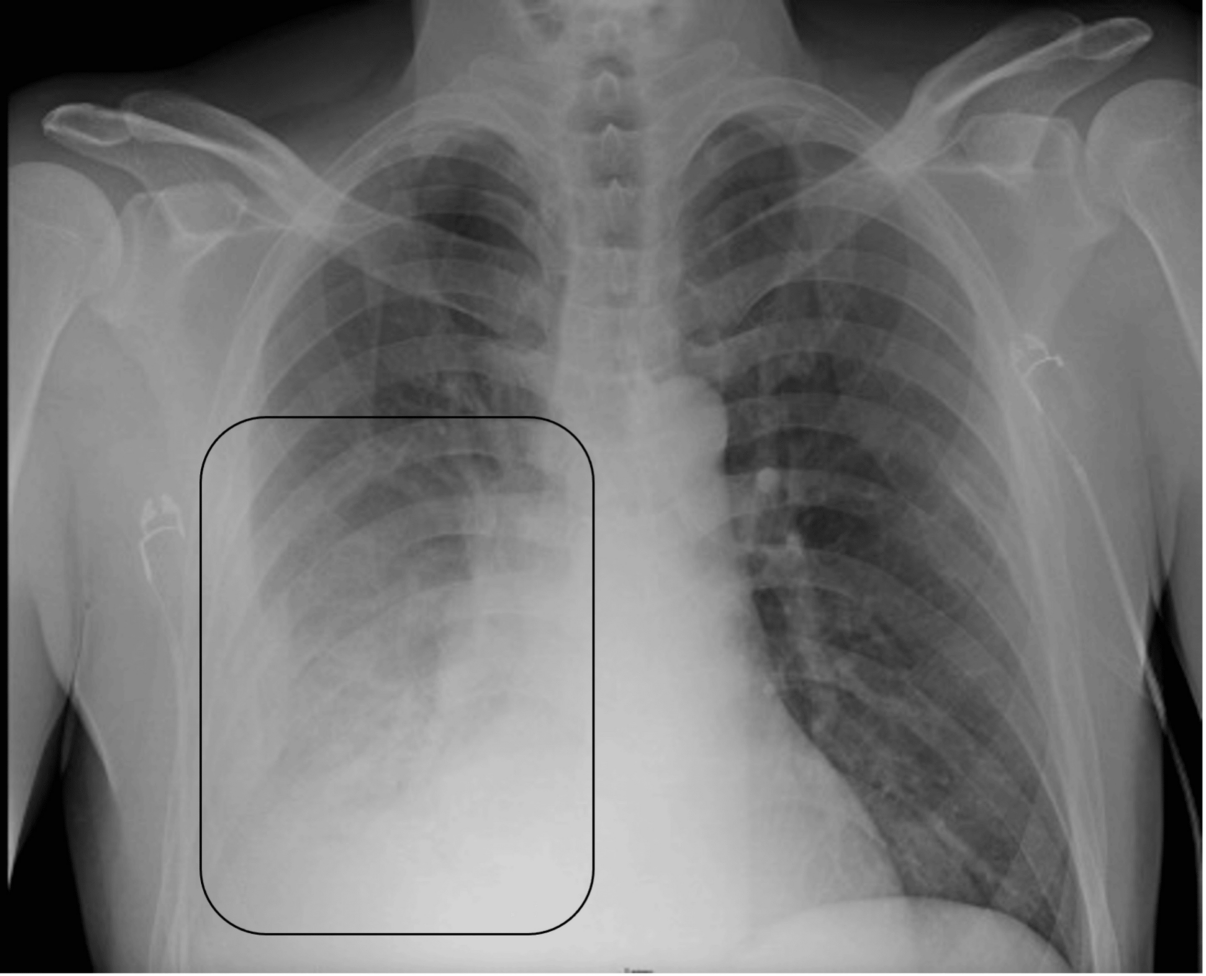
Chest X-ray revealing a loculated right pleural effusion accompanied by right lower lobe consolidation, clearly marked within the black square. The image demonstrates the separation of pleural fluid and lung consolidation, indicating a complex infectious process in the right hemithorax.

**Figure 2:**
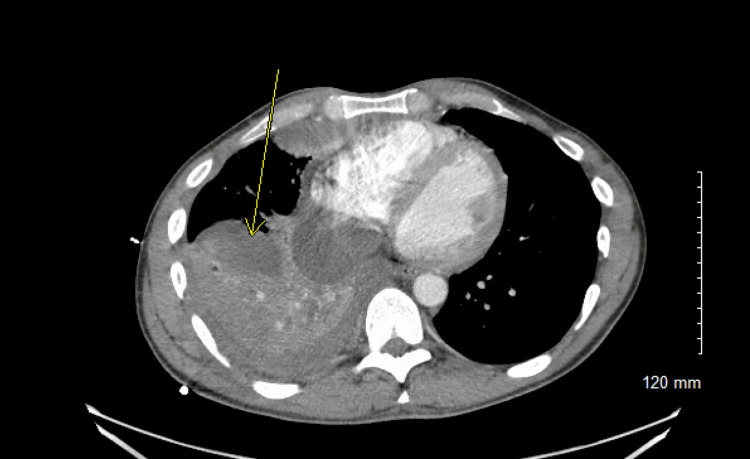
CT chest with IV contrast, axial view, showing a multiloculated right pleural effusion as indicated by the yellow arrow. The image highlights the complex fluid collection within the pleural cavity, demonstrating multiple compartments and septations consistent with advanced empyema.

**Figure 3:**
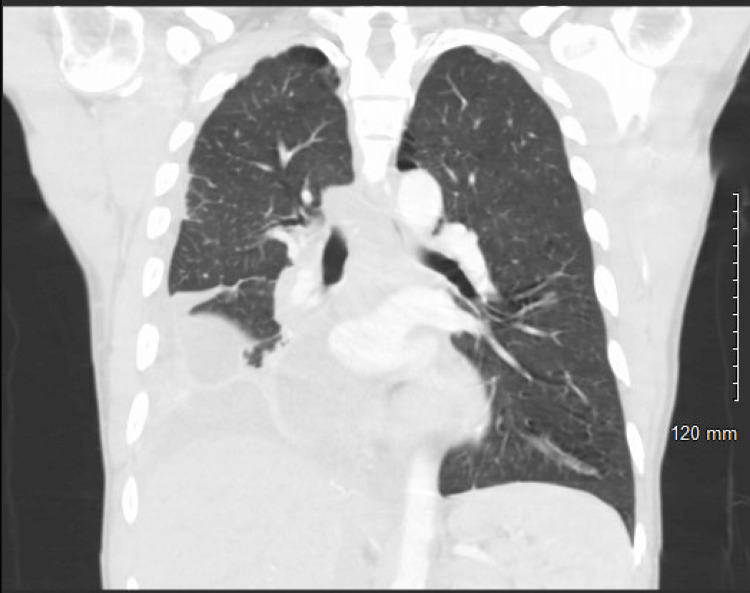
CT chest lung window, coronal view shows multiloculated pleural effusions

Given the patient's presentation with acute respiratory failure, hypoxia, and right-sided chest pain, imaging was immediately warranted to assess the underlying cause. Initial imaging included a chest X-ray (Figure 1) followed by a CT scan (Figures 2, 3) to evaluate the extent of the suspected infection and structural abnormalities. The CT scan revealed right-sided consolidations and a loculated pleural effusion. Laboratory tests indicated elevated white blood cell count (WBC), hemoglobin, hematocrit, and platelets, suggesting a significant inflammatory response. Imaging findings consistently indicated right lower lobe pneumonia, atelectasis, and persistent loculated fluid collections despite chest tube placement. Empiric antibiotics were initiated with azithromycin and ceftriaxone, which were later escalated to vancomycin and piperacillin/tazobactam due to the persistence of fever and tachycardia.

Consultations were initiated with cardiothoracic surgery, pulmonology, nephrology, and infectious disease specialists. A chest tube was placed to drain the pleural effusion, and tPA plus dornase were administered to improve drainage. Despite these measures, daily chest tube output remained elevated (approximately 300-500 mL), and follow-up imaging confirmed incomplete lung re-expansion alongside multiple loculated fluid collections. Although the patient reported subjective improvement, the persistently serosanguineous drainage prompted cardiothoracic surgery to recommend surgical intervention.

**Figure 4:**
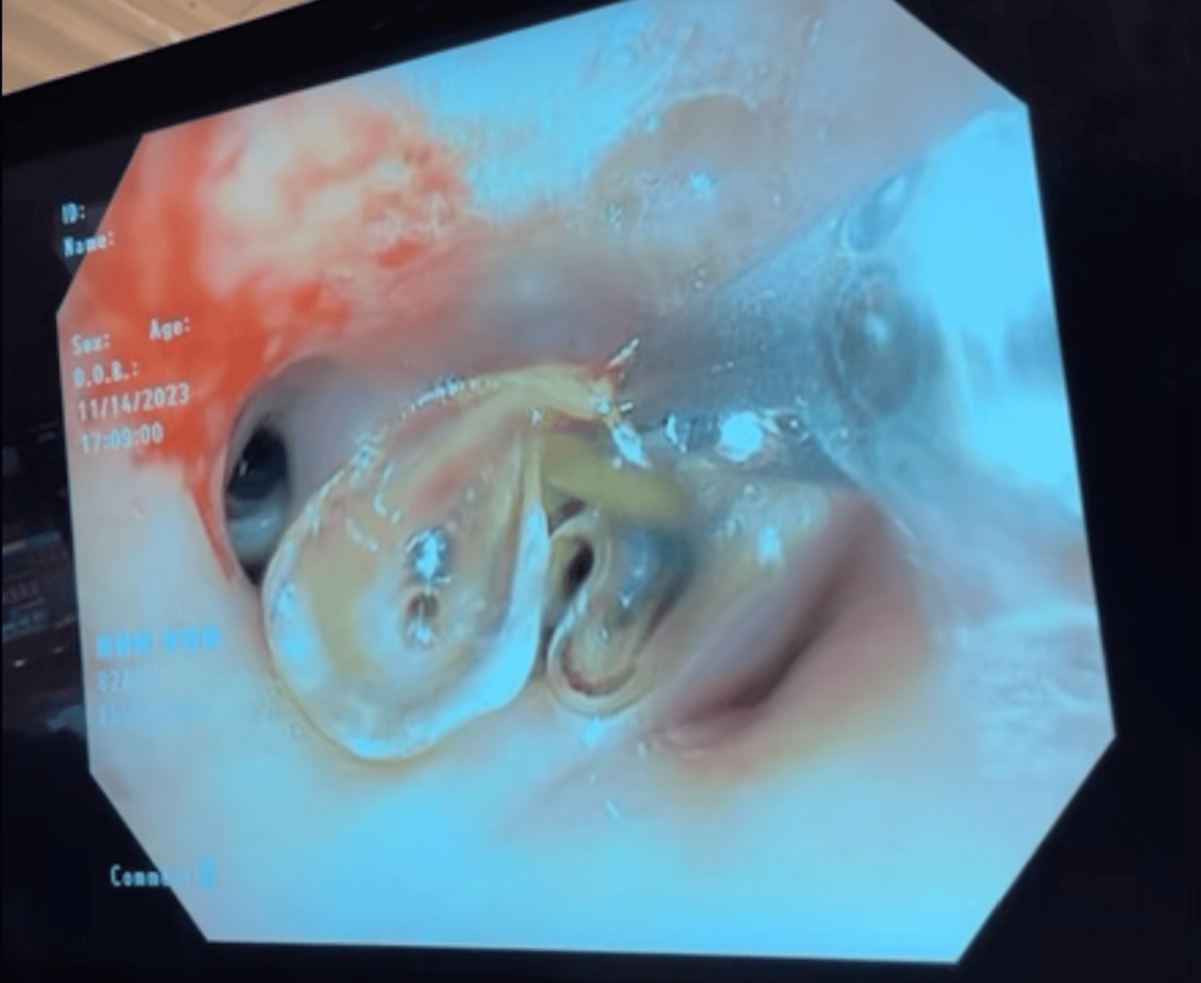
Bronchoscopic view showing the extraction of a foreign body impacted in the right lower lobe, later identified as a fentanyl patch. The image captures the retrieval process, highlighting the obstructive foreign material within the bronchial tree.

**Figure 5:**
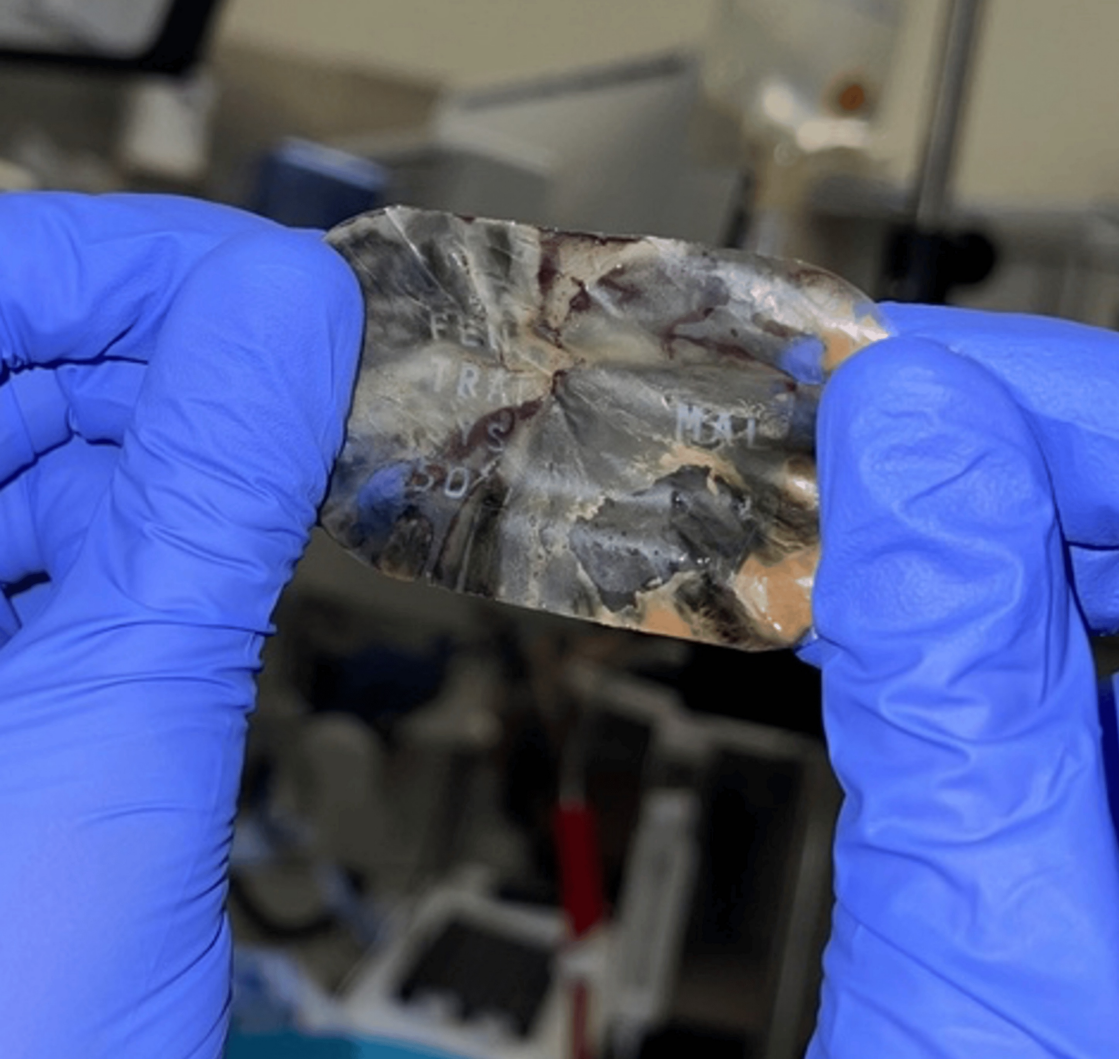
The extracted foreign body, identified as a fentanyl patch, which the patient had aspirated unknowingly for an unknown period. The image clearly shows the patch post-extraction, providing visual evidence of the obstruction responsible for the patient's severe pulmonary complications.

The patient underwent right thoracoscopy, which was converted to an open thoracotomy with decortication due to dense adhesions. During surgery, bronchoscopy was performed, revealing significant secretions and a foreign body (fentanyl patch) in the right lower lobe (Figure 4). The fentanyl patch was removed (Figure 5), releasing a substantial amount of purulent material. The decortication resulted in good re-expansion of the right lower lobe.

Postoperatively, the patient demonstrated steady improvement, with decreased oxygen requirements and the ability to ambulate. Radiological evaluation indicated sufficient lung re-expansion, supporting the decision to remove the chest tubes. Infectious disease specialists recommended transitioning to oral amoxicillin/clavulanate for continued antibiotic therapy. However, the patient developed elevated creatinine levels, suggesting acute tubular necrosis (ATN), which nephrology managed appropriately. He was ultimately deemed stable for discharge with planned outpatient follow-up to monitor renal function and pulmonary status.

**Figure 6:**
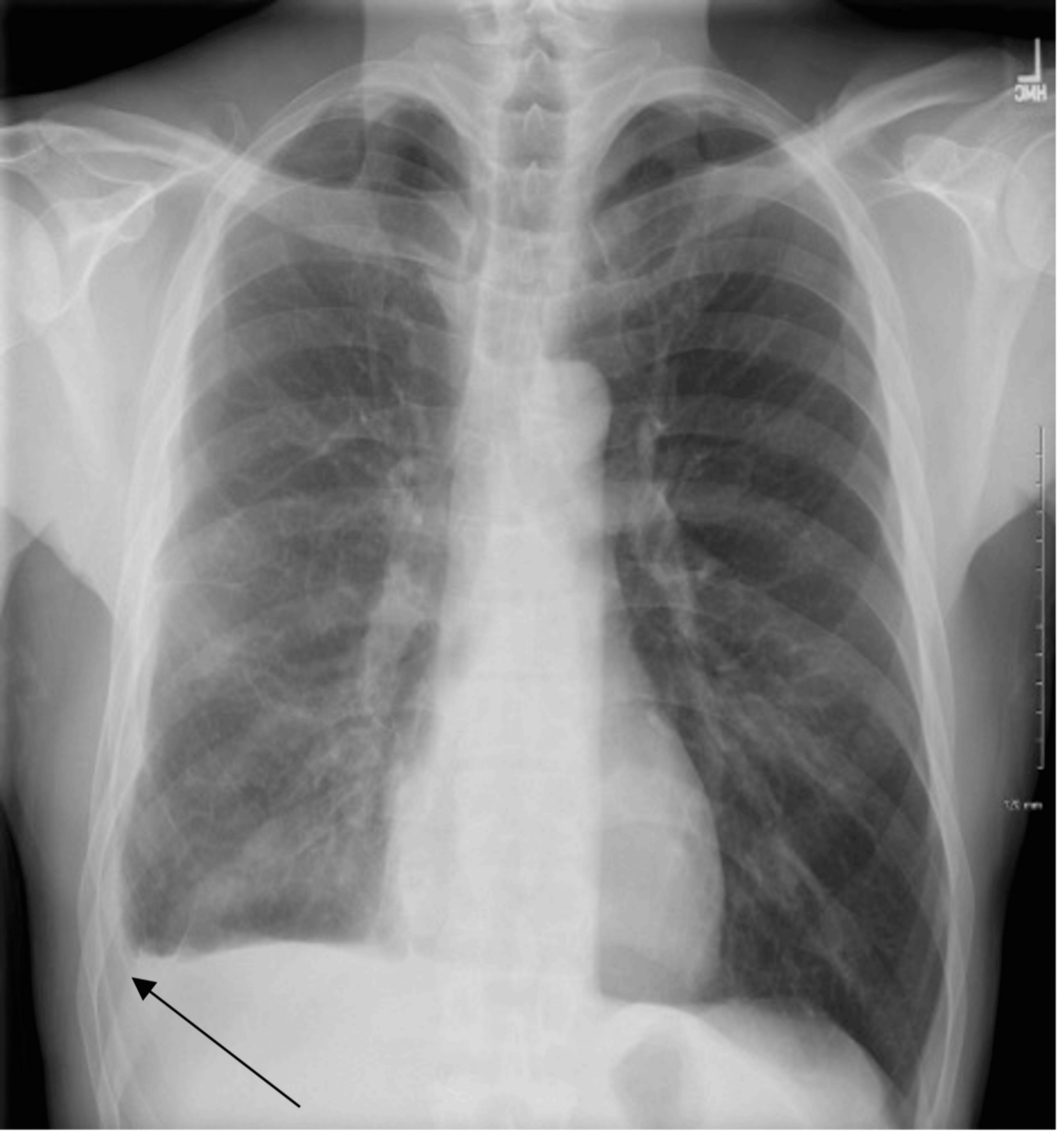
Chest X-ray displaying blunting of the right costophrenic angle (indicated by the black arrow) with significant improvement in the right sided pleural effusion. The image demonstrates reduced pleural fluid and partial resolution of the previous consolidation, indicating a positive response to treatment.

Following discharge, the patient’s symptoms improved significantly. His chest X-ray showed significant improvement of the right lower lobe effusion (Figure 6). He was advised to use an albuterol metered-dose inhaler (MDI) as needed for shortness of breath, undergo pulmonary function tests (PFTs), and stop chewing tobacco, having already quit smoking.

From further discussions with the patient during the follow-up period, he admitted to applying the transdermal patch to his palate and believes he accidentally aspirated it while unconscious. This incident likely occurred around five years ago, correlating with his recurrent episodes of pneumonia. This information provided a crucial insight into the chronic nature of his respiratory symptoms and highlighted the importance of thorough patient history and follow-up in managing complex cases.

## Discussion

Empyema often develops from parapneumonic effusions or, less frequently, from conditions like lung abscesses or post-surgical infections [11]. Effective management typically requires timely diagnosis, targeted antibiotic therapy, and, when necessary, surgical intervention to achieve complete resolution [15]. The situation becomes even more complex when a foreign body is involved, as seen in this case, where a fentanyl patch served as the driving force behind persistent infection and pleural involvement.

The combination of acute respiratory failure, hypoxia, and right-sided chest pain prompted an urgent and comprehensive evaluation. Chest X-rays and CT scans demonstrated right-sided consolidations and a loculated pleural effusion, findings suggestive of an empyema. Laboratory tests revealed elevated white blood cell count, hemoglobin, hematocrit, and platelets, further indicating a pronounced inflammatory process. Empiric antibiotic therapy was initiated, and once preliminary culture results became available, the regimen was adjusted to vancomycin and piperacillin/tazobactam in response to the suspected pathogens.

Placing a chest tube to drain the pleural effusion was a critical initial step. Although the use of tPA and dornase can produce sanguineous drainage, follow-up imaging continued to show multiple loculated fluid collections and incomplete lung re-expansion. These findings indicated that standard measures alone were not sufficient, prompting the need for surgical washout and decortication to fully resolve the infection and restore adequate pulmonary function.

The decision to proceed with surgical intervention was based on the persistent loculated effusion and the patient's overall clinical status. The patient underwent a right thoracoscopy, which was converted to an open thoracotomy with decortication due to the presence of dense adhesions.

A bronchoscopy was performed intraoperatively due to the extensive adhesions observed in the right lower lobe and the suspicion of an additional obstructive process. This procedure proved pivotal in identifying the root cause of the empyema. The bronchoscopy revealed copious secretions and a fentanyl patch lodged in the right lower lobe. Removing the foreign body led to a marked release of purulent material, confirming the patch’s central role in perpetuating the chronic infection and inflammatory response.

Postoperatively, the patient demonstrated significant improvement. Oxygen requirements decreased, and subsequent chest X-rays showed resolution of the major abnormalities. The patient’s ability to ambulate and improved clinical status facilitated the transition from intravenous to oral antibiotics, with Augmentin prescribed based on infectious disease recommendations.

This case shows bronchoscopy’s vital role in diagnosing and treating post-obstructive pneumonia that contributed to the development of empyema, particularly when a foreign body is involved. Identification and removal of the fentanyl patch during bronchoscopy relieved the obstruction, reduced ongoing inflammation, and prevented further infectious complications, leading to a marked improvement in the patient’s clinical course. It also reinforces the importance of maintaining a high index of suspicion for foreign bodies in patients with unexplained or persistent pulmonary infections, especially those with a history of substance abuse.

The discovery of a fentanyl patch as the foreign body in the patient’s right lower lobe, aspirated and left unnoticed for an unknown length of time, is exceptionally rare. Such a finding underlines the need to consider foreign body aspiration in patients presenting with persistent or unexplained respiratory issues, particularly those with a history of substance abuse.

Foreign bodies in the lung can lead to a variety of complications beyond empyema, such as pneumonia, bronchiectasis, lung abscesses, and obstructive atelectasis [16], as outlined earlier. Chronic inflammation and infection may severely impair lung function, promote recurrent respiratory infections, and potentially culminate in sepsis or respiratory failure [17]. The obstruction itself can compromise ventilation in certain lung segments, raising the likelihood of more severe outcomes [18].

Empyema is relatively uncommon, with an estimated incidence of three to six cases per 100,000 persons annually in the United States [19]. It is more frequently observed in children and the elderly, often arising as a complication of bacterial pneumonia, thoracic surgery, or an inadequately drained hemothorax following chest trauma [20].

Empyema management generally entails a multidisciplinary approach, incorporating antibiotic therapy, pleural drainage, and, in selected cases, surgical intervention [20]. The initial step often involves administering broad-spectrum antibiotics tailored to culture findings [21], followed by pleural drainage via chest tube placement. Intrapleural fibrinolytic therapy, such as tPA combined with dornase alfa, has been supported by studies (e.g., the MIST2 trial) to enhance drainage by breaking down loculations, which can decrease the need for surgical procedures [22]. In more refractory or severe cases, surgical interventions like VATS or open thoracotomy with decortication may be required to remove the fibrous pleural peel and fully evacuate the infected material [23].

Empyema can give rise to complications such as sepsis, respiratory failure, and pleural fibrosis, especially when diagnosis is delayed or in patients with pre-existing conditions [24]. These adverse outcomes often emerge during prolonged disease courses or when initial management is insufficient, and they can significantly increase hospital stays and healthcare costs [20]. The mortality rate for empyema varies but may reach 20% in specific populations, including older adults and individuals with multiple comorbidities [25]. Furthermore, empyema can amplify underlying health issues, leading to poorer overall outcomes and a higher risk of mortality [26].

The patient’s aspiration of a fentanyl patch likely took place during a period of substance abuse, where reduced airway protection and impaired consciousness make foreign body aspiration more probable. Post-obstructive pneumonia may develop due to fluid stasis and bacterial overgrowth behind the obstructing object, potentially progressing to empyema or even a lung abscess if left untreated [27]. Management in such cases involves targeted antibiotic therapy, removal of the foreign body to re-establish airway patency, and, when indicated, drainage or surgical intervention for any resulting complications. Delayed or inadequate treatment can lead to prolonged hospitalization, increased morbidity, and higher rates of adverse outcomes.

In this case, the aspirated fentanyl patch led to recurrent pneumonia due to prolonged bronchial obstruction, ultimately progressing to empyema. This emphasizes the need for detailed diagnostic evaluations in patients with persistent respiratory symptoms and a history of substance abuse [28]. A multidisciplinary approach incorporating comprehensive imaging, bronchoscopy, surgical intervention for drainage and decortication, and coordinated medical care proved pivotal in resolving the chronic infection and ensuring a favorable outcome for the patient [29].

## Conclusions

Foreign body aspiration is a rare but serious cause of pleural infections such as empyema, particularly in patients with a history of substance abuse. Effective management often requires a multidisciplinary approach, including comprehensive diagnostics, antibiotic therapy, and surgical intervention. Bronchoscopy is crucial for both diagnosis and removal of the foreign body. Awareness and prompt identification of foreign body aspiration can significantly improve patient outcomes and reduce complications.
